# Velpeau on Cysts of the Eyelid

**Published:** 1842-10

**Authors:** 


*M. Velpeau on Cysts of the Eyelid.*—I would say a few words respecting the case of a man, on whom I am about to operate for three small tumors on the eyelid. Of two of these the outline cannot be distinguished, being masked by the third which is more voluminous. This last is evidently a cyst filled with a fluid or semifluid material. The nature of the contents of the two others it is not easy to diagnose, from their size and the thickness of their walls, but from analogy we may suppose them to be filled with a liquid or semiconcrete matter.

Tumors of this description are very rarely cured by any plan of treatment except operation. Occasionally, indeed, they disappear without any operation or treatment. Sometimes this may result from the particular constitution of the individual, as in a young subject who about the age of puberty was freed from an affection of this nature. Or, it may be produced by some other disease concurring at the same time, as in the case of a woman who had three of these tumors on the eyelid, and on whom I had fixed a period for operation. But she came to me some months afterwards, and informed me that she had suffered under an affection of the chest, and the tumors had disappeared. Occasionally they disappear under local applications for their resolution, but we can rarely expect success to attend this treatment, and it is altogether ineffectual when they have attained a considerable developement. They may, however, be safely and with certainty cured by surgical means.

Of the two proceedings, extirpation and incision, the former, employed by most surgeons, appears to me a tedious operation. It is necessary to dissect with caution, and if it be a cyst, whatever care is taken, it will be difficult to avoid opening it; it then is necessary to excise it entirely, and to apply cauterization, or there will be a considerable chance of return. If the contents of the tumor be concrete, the operation is comparatively easy; but, otherwise, I think incision is preferable: it is less painful, and quite as successful. Some persons in practising this operation hold the tumor with the fingers, others place an elevator beneath the eyelid. I employ simply two pair of forceps, of which one is held by an assistant; I myself, holding the other in such a manner that the eyelid is rendered tense between them, and raised from the globe of the eye. I make the incision, expel the contents, and apply nitrate of silver to the interior of the cavity. There is sometimes slight consecutive inflammation, and the eyelid swells a little, and soon an eschar is detached and the cure accomplished in eight days. A piece of lint dipped in saturnine lotion or cold water is the only dressing employed. I should, however, observe, that by extirpation the cure is more prompt; a piece of dressing is placed on the wound, and the whole is terminated.

After these observations the operation was performed: M. Velpeau incised the different tumors and cauterized the bottom of each wound.—*Lond. Med. Gaz., from a Clinical Lecture by M. Velpeau, in Gaz. des Hopitaux.*

